# Studying modulation on simultaneously activated SSVEP neural networks by a cognitive task

**DOI:** 10.1007/s10867-013-9335-7

**Published:** 2014-01-13

**Authors:** Zhenghua Wu

**Affiliations:** 1 School of Computer Science and Engineering, University of Electronic Science and Technology of China, Chengdu, 610054 China; 2 Key Laboratory for Neuro Information of Ministry of Education, School of Life Science and Technology, University of Electronic Science and Technology of China, Chengdu, 610054 China; 3 Department of Biomedical Engineering, University of Florida, Gainesville, FL 32611 USA

**Keywords:** Steady-state visually evoked potential (SSVEP), Steady-state probe topography (SSPT), SSVEP neural network, Cognitive neural network

## Abstract

Since the discovery of steady-state visually evoked potential (SSVEP), it has been used in many fields. Numerous studies suggest that there exist three SSVEP neural networks in different frequency bands. An obvious phenomenon has been observed, that the amplitude and phase of SSVEP can be modulated by a cognitive task. Previous works have studied this modulation on separately activated SSVEP neural networks by a cognitive task. If two or more SSVEP neural networks are activated simultaneously in the process of a cognitive task, is the modulation on different SSVEP neural networks the same? In this study, two different SSVEP neural networks were activated simultaneously by two different frequency flickers, with a working memory task irrelevant to the flickers being conducted at the same time. The modulated SSVEP waves were compared with each other and to those only under one flicker in previous studies. The comparison results show that the cognitive task can modulate different SSVEP neural networks with a similar style.

## Introduction

When a visual stimulus with a steady frequency such as a flicker or a reversal pattern appears in the visual field of a person, an electrical signal can be observed via the scalp, with the same frequency as that of the stimulus. This signal is referred to as steady-state visually evoked potential (SSVEP) [1,2]. However, the mechanism of SSVEP is not yet clear. The term ‘network’ is used in many different fields including communication, social science, and neuroscience. When used in neuroscience, this term is traditionally used to refer to a network of biological neurons that can be activated together to complete a special function [2–4]. For different cognitive functions of the brain, it is often explained based on different cognitive neural networks [4–7]. In studying the mechanism of SSVEP, neural network theory is adopted and a group of neurons in the cortex activated together by a flicker considered an SSVEP neural network [8–10]. One theory suggests that SSVEP is a cortical response via cortico-cortico loops to the stimulus presented to the peripheral retina and there exist three SSVEP neural networks corresponding to different frequency bands, i.e., low (5–12 Hz), medium (12–30 Hz), and high (30–50 Hz) frequency [2]. Some other studies have demonstrated surely that resonance exists in these bands [6,8]. This theory is widely accepted in many SSVEP studies [3–5,7,11,12]. Another study found that a flicker in a wide frequency range from 1 to 100 Hz or so can evoke SSVEP with resonance peaks around 10, 20, 40, and 80 Hz [9]. Unlike cognitive neural networks, probably located in different brain areas for different tasks, SSVEP neural networks are thought to be primarily located in the cerebral cortex [2,10]. Although the powers of different frequency SSVEP are very different from each other, all of them concentrate at the occipital area, and the SSVEP in other areas are thought to transfer from the occipital area. In other words, the SSVEP neural networks can be more like locative networks. While for a cognitive neural network, normally many brain areas can be involved simultaneously. A certain brain area can be involved in a cognitive neural network; it can also be involved in other cognitive neural networks. For example, the primary vision cortex can be involved in a memory or visual vigilance task [4,10]. So, normally, cognitive neural networks are more like global networks. When a cognitive neural network related to the primary vision cortex is activated, an SSVEP neural network is also activated at the same time. Because they are both related to the primary vision cortex, there can be some interaction between the two networks. Specifically, the recognitive task modulates the SSVEP. From studying modulated SSVEP waves, the recognitive process can be understood.

Since SSVEP was discovered, it has been used in many areas. One important area concerns the brain–computer interface (BCI) [11–16], in which many flickers with different frequencies are used to stimulate the subject’s eyes. By recognizing the frequency components in the subject’s SSVEP, the flicker that the subject is staring at is determined, then the task corresponding to this flicker can be finished automatically. Another important use is in studies related to the process of a cognitive task of long duration [5,17–20]. When an event such as a visual stimulus happens in a short period, a signal called an event-related potential (ERP) can be observed from the scalp of the subject. An ERP in a short period can be extracted using the superposition method. In this method, the ERP for each trial is hypothesized to be synchronous with the event and similar between trials. After superposing all EEGs in every trial together, the synchronous ERP is strengthened, while the other background EEG components can be canceled almost for their asynchronism. However, for a task lasting for a long period, this superposition method is not valid because the disturbance in different trials is unique and cannot be canceled completely by superposition. As we know, the fast Fourier transform (FFT) method is often used in extracting SSVEP [7,21–25]. By selecting the integration period carefully, a suitable frequency resolution can be obtained. If subjects are stimulated by a repetitive flicker when they are performing a cognitive task of long duration, for example, longer than 10 s, some researchers suggest that the combination of the short Fourier integration period and the extracting ERP technique maximizes the possibility of observing SSVEP changes associated with the cognitive processes. Furthermore, it has also been suggested that the SSVEP amplitude and phase changes can be used to explain the cognitive process. This technique is referred to as steady-state probe topography (SSPT) [4] and will be detailed in the methodology section.

Since the introduction of the SSPT method, many works based on the method have been done. In these SSPT-based studies, only one SSVEP neural network, i.e., the middle frequency band neural network, was activated during the cognitive task [17,19,24] and none have discussed the validity of popularizing this method to a wide frequency band. In 2007, in order to know whether the modulations on the different SSVEP neural networks by the same task were identical, we used two flickers to separately activate the different SSVEP neural networks. At the same time, the subject was asked to do the same cognitive task [26]. Comparing the two modulated SSVEP waves obtained in that experiment showed some consistency of the modulation on different SSVEP neural networks by the same task. There was, however, still a clear discrepancy in some situations. This discrepancy may be caused by background noise and potential psychological activity.

We hypothesized that if two SSVEP neural networks and one cognitive neural network were activated simultaneously by two different frequency flickers and one cognitive task respectively, the cognitive neural network would affect the two SSVEP neural networks. In other words, the response to the cognitive task interacts with the response to the flickers. Because the influence on the two SSVEP neural networks by the background noise or potential psychological activity are more similar to each other than in previous works, the interactions between cognitive neural network and SSVEP neural network can display a higher consistency than in previous studies. This suggests that the mechanism of interaction between the cognitive neural network and the SSVEP neural network is definitive.

In order to test the above hypothesis, in this work two flickers with different frequencies were utilized simultaneously to stimulate the subject (one was 8.3 Hz, corresponding to the SSVEP neural network in the low band, and the other was 20 Hz, corresponding to the SSVEP neural network in the medium band). At the same time, a cognitive task that continues for 12 s in each trial was conducted by the subjects. A 129-channel EGI was used to collect the EEG signal. The SSPT method was used to analyze the amplitude and phase of SSVEP. The results indicate that the cognitive neural network modulates the different SSVEP neural network in a similar way.

## Methodology

### Stimulus design

This study was approved by the Human Research and Ethics Committee of the University of Electronic Science and Technology of China. Before the experiment, all the subjects were told the purpose and procedure of the experiment in detail and signed a consent form. These forms were sent to the Ethics Committee of our university and approved.

All ten subjects involved in this experiment were either normal sight or corrected normal sight. In order to compare with our previous study in which the subjects were all males, only male subjects were selected. The mean age was 26 (range 26 ± 2) years old. Two white high-luminance LED flickers were attached to the computer screen at a horizontal angle of 0° and a vertical angle of 35° up to the center of screen. The two LEDs had a diameter of about 0.5 cm and were placed close to each other, serving as the stimulators of SSVEP and were driven by two square waves with different frequencies (one being 8.3 Hz and the other 20 Hz). These two square waves were generated by a pulse signal generator. The pulse duration of the high and low level can be set separately. In this study, the duty cycle was set to 1, i.e., the duration of the high level and low level was the same. For the 20-Hz stimulator, the cycle was 50 ms, so the duration of the high and low levels were 25 ms, respectively. For the 8.3-Hz stimulator, the cycle was 120 ms, so the duration of the high level and low levels were 60 ms. For the subject’s comfort during the experiment, they were seated in a chair, the height of which could be conveniently adjusted. The distance from the screen was 60 cm. The experiment was divided into two stages, i.e., the preliminary experiment and the formal experiment. In the preliminary experiment, each LED was driven separately for 64 s and the SSVEPs during this period were recorded for comparison with those that were evoked simultaneously by two flickers. In the formal experiment, the two flickers were driven simultaneously, with the subjects being required to perform a graded working memory task shown on the screen.

The graded working memory task was similar to that of Silberstein and our previous work [10,26] and there were three classes of task in this experiment. The first class with five stages is called the low demand task (LD). In the first stage (prepare stage) of LD, a cross surrounded by a circle is displayed in the center of the screen for 2 s, with the cross being used to attract the attention/fixation of the subject. In the second stage (intake stage), one irregular polygon and three filled circles are displayed in the four quadrants of the cross for 2 s and the subjects asked to remember the shape and position of the irregular polygon. In the third stage (hold stage), the subjects are required to memorize the information received in the second stage while a single cross is displayed for 4 s. During the fourth (probe stage), a probe figure, an irregular polygon in a quadrant of a cross, is displayed and the subjects asked to judge whether the shape and position of the irregular polygons are the same as those shown in the second stage. If they are the same, button ‘1’ is pushed; otherwise button “3” is pushed. All the judgments needed to be completed within 2 s. In the fifth stage (recover stage), only a black screen is displayed for 2 s and the subjects are allowed to relax during this period.

The second class task is the high demand task (HD) and is the same as LD, except that there are two irregular polygons and two filled circles in the four quadrants of a cross. The time sequence of the HD or LD includes the ‘prepare’ stage (2-s), ‘intake’ stage (2-s), ‘hold’ stage (4-s), ‘probe’ stage (2-s), and ‘recover’ stage (2-s). The third class is the control task (C). Under the ‘C’ task, only one irregular polygon is used, there is no ‘hold’ stage after the ‘intake’ stage and a ‘probe’ stage starts immediately to provide quick, correct discrimination. In order to compare to the ‘LD’ and ‘HD’ classes, the first stage in the ‘C’ task is set to 2 s, the second stage (‘delay’) is set to 4 s, in which the subjects simply hold their fixation on the center of the screen and the third stage is the ‘intake’ stage of 2 s. The time sequence of ‘C’ includes the ‘prepare’ stage (2 s), ‘delay’ stage (4 s), ‘intake’ stage (2 s), ‘probe’ stage (2 s), and ‘recover’ stage (2 s).

**Fig. 1:**
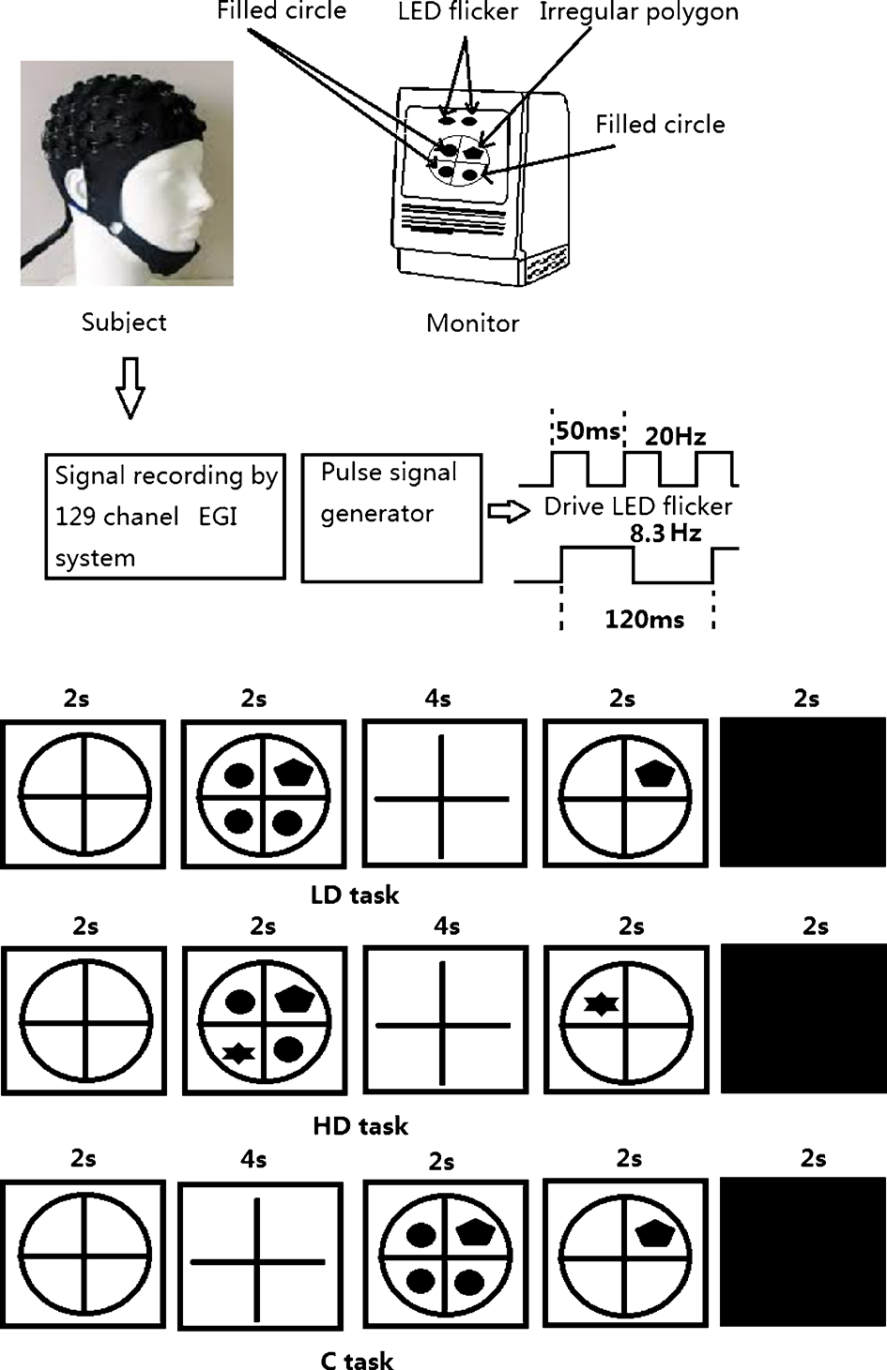
The experimental paradigm and the stimulus setup. The outputs of the pulse signal generator are connected to the LEDs

The memory task includes 32 continuous LD trials, 32 continuous HD trials, and 32 continuous C trials. Each trial lasts for 12 s, so each class task lasts 384 s (12*32) as a block. Between each block, subjects can rest. For different subjects, the run order is different, i.e., the run order is counterbalanced within the subjects. Figure 1 displays the experimental paradigm and the stimulus setup.

### EEG recordings

**Fig. 2:**
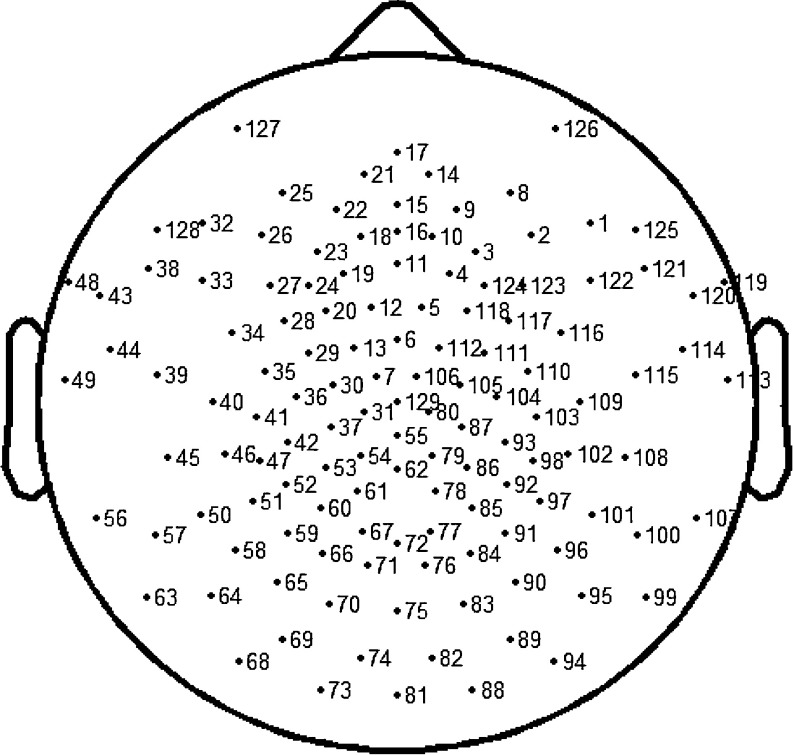
The electrode map of the 129-channel EGI system

The recordings were made using a ‘Net Amps 200’ amplifier with a 129-channel electrode cap (EGI system 200, Electrical Geodesics Incorporated, USA). The 129 channels included 128 measuring electrodes and one reference electrode Cz and the international 10-20 recording location was included in this system. Electrode impedance was kept below 10 kΩ, in order to retain good contact with the scalp and salt water dropped into the electrode periodically. The sample rate was 250 Hz, with the EEG being sampled every 4 ms and the sampled signal filtered by a band-pass of 0.3–45 Hz and stored on a disk for off-line analysis. All the experiments were performed in a shielded room with the door and window closed to filter outside noise. Figure 2 shows the electrode map of the EGI system.

### Data analysis

Before being processed with the SSPT method, the EEG was preprocessed. The preprocessing method employed in this work is simple. The EEG of each subject is first visually inspected. Any recording that exceeds 100 *μ*v is identified as a failure and replaced by the mean of its three nearest neighboring recordings around the electrode. Except for this artifact removing method, no other methods are adopted. The frequency of noise such as eye movement is normally low and far from the flicker frequency. This has little influence on the power of SSVEP frequency. Only the power and phase of SSVEP frequency are utilized in the SSPT method. Therefore, no other preprocessing methods such as eliminating eye movement are employed. This preprocessing method is employed widely in works based on the SSPT method for studying recognitive tasks such as memory and emotion [4,5,7,10,17–19]. The signal processing technique SSPT described below are based on the fast Fourier transform (FFT), in which, if the data volume is large enough, a high-frequency resolution can be obtained and the artifact impact such as eye movement can decrease significantly. The SSPT method is only used to extract the power and phase of SSVEP frequency, not to extract directly the response of the cognitive network modulated by the task. In fact, the preprocessing adopted in the SSPT method is aimed at removing the noise in the same band as the SSVEP frequency, not noise far from SSVEP frequency such as eye movement. We have compared SSVEP in trials, with and without ocular artifacts, with no significant difference being discovered. If big noise appears at more than three contiguous electrodes, the preprocess begins from the edge of this area, and reconstructs the signal at each electrode one by one. Of course, if there are too many of these situations, it would decrease the truthfulness of the SSVEP waves. In a lab environment, the conditions are strictly controlled; and rarely is big noise produced within multiple contiguous electrodes. In this work, this situation did not appear. Because of the high signal-to-noise ratio of SSVEP to noise such as eye and body movement, in the SSVEP-based BCI [11–13,16,23], normally there is no preprocess method adopted.

All the main data analysis methods were the same as those in our other similar work [26] and in order to make it easy to understand the process of analysis, these methods are re-described below. The trial under which the subject reacts incorrectly is rejected and the average reference is selected as the re-reference before analyzing the EEG data. The SSPT method is adopted to estimate the amplitude and latency of the SSVEPs, which can be divided into four steps as described.

Step 1:    A sliding window with a length of 1.2 s is used to sample the SSVEP data in each channel. For the data in each sliding window, the amplitude and phase of 8.3 Hz and 20 Hz is calculated by FFT, with a temporal resolution of 1.2 s and a frequency resolution of 0.83 Hz (1/1.2). The shifting step of the sliding window is 12 ms. This process is continued until all the valid EEG data are analyzed and finally we get a 12-s SSVEP sequence (including amplitude and phase) for each trial.
Step 2:    For the HD, LD, and C trials, the 12-s epochs of SSVEP (amplitude and phase) obtained in step 1 were averaged over the trials. This yielded 129 channels of SSVEP amplitude and phase with a 12-s length for each task. The resulting 129-channels amplitude in the C task were further averaged across the 129 channels to get a unique baseline SSVEP amplitude, named as the normalization factor (NF). The 129-channel amplitude of the HD, LD, or C task were then divided by the NF to get a normalized amplitude and the processes applied to each subject. The normalized amplitude was further averaged across all subjects and the final group averaged normalized amplitude, with a size of 12 s and 129 channels. This normalized amplitude is thought of as an indicator of the response intensity for the HD or LD task compared to the C task and the higher this value is, the stronger the response of the corresponding cognitive neural network [4,5,10,17].
Step 3:    For the phase that represents the latency of SSVEP, the result show a wide variation among all the electrodes, thus it is not appropriate to define a unique phase NF for each subject by averaging the phases over channels. In this work, the phase difference between C and HD or C and LD at each electrode is averaged across all subjects, and these phase differences are shown as latencies (ms) using the formula:
  $$\left[ {\frac{{\mathbf{change}} ~ {\mathbf{in}} ~ {\mathbf{phase}}}{\left( {\mathbf{2}\ast \prod } \right)}} \right]\ast \left( {\frac{{\mathbf{1000}}}{{\mathbf{stimulus}} ~ {\mathbf{frequency}}}} \right),$$

where the ‘change in phase’ means the phase difference in radian between C and HD or between C and LD. The ‘1000/stimulus frequency’ indicates the flickering cycle in milliseconds. In works based on the SSPT method, the phase difference (or latency) is thought of as an indicator of the response speed for the HD or LD task compared to the C task. If this value is negative, it suggests that the cognitive neural network reacts slower for HD or LD tasks than for C tasks and vice versa [4,5,10,17].

Step 4:    SSVEP amplitude and latency are displayed in topographic maps using the software EEGLAB. A one-way analysis of variance (ANOVA) is performed to check the difference of the SSVEP power under different situations and the interaction between the two SSVEP neural networks. The significance level ‘*p*’ of the ANOVA result is set to 0.05. If the ‘*p*’ value is smaller than 0.05, it suggests that there is a significant difference between the two compared situations. Additionally, a correlation coefficient is used to estimate the similarity of modulated SSVEP waves or the SSVEP power distributions. The ANOVA is finished by the function ‘anova1’ in ‘MATLAB’ software. For example, for subjects S1, S2,... S10 in this work, when stimulating at 8.3 Hz, the average SSVEP power across all 129 electrodes is a1, a2,... a10, respectively, when stimulating at 20 Hz, it is b1, b2,... b10, respectively. Although the difference between the 8.3-Hz and 20-Hz SSVEP power is different for different subjects, in general, the 8.3-Hz SSVEP is bigger than that of the 20-Hz SSVEP. In order to understand the difference in significance between the 8.3-Hz and 20-Hz SSVEP, ANOVA can be applied to these two series. If the ANOVA ‘*p*’ is smaller than 0.05, it suggests that the 8.3-Hz SSVEP is significantly bigger than that of the 20-Hz SSVEP. The SSVEP power distribution can be understood via a series of 129 data lengths. In order to understand the difference in significance of SSVEP power distribution under different situations, it is necessary to find a parameter standing for the property of the power distribution series. The autocorrelation function of a series can stand by the relationship between the points, so the sum of the autocorrelation coefficient of the power distribution series is used as an indicator of SSVEP power distribution. Then, ANOVA can be applied on this SSVEP power distribution indicator.

## Results

The accuracy of the ‘C’ task is the highest and all ‘C’ tasks are recognized correctly by every subject. The average accuracy of ‘HD’ is about 92% (average 29.5 trials of 32 are correctly recognized) and the average accuracy of ‘LD’ is above 99% (only three trials of the total 320 being incorrectly recognized). The SSVEPs at all electrodes have been computed, and the ANOVA about SSVEP power is based on the average power across all electrodes, while the ANOVA about SSVEP distribution is based on the power distribution indicator. In showing the modulated SSVEP waves, the waves at electrode No. 60 (near the Pz in the 10–20 system) are selected to compare with the results of previous work [26] in which this electrode was chosen as an example.

### SSVEP power and distribution under a situation with no cognitive tasks

**Fig. 3:**
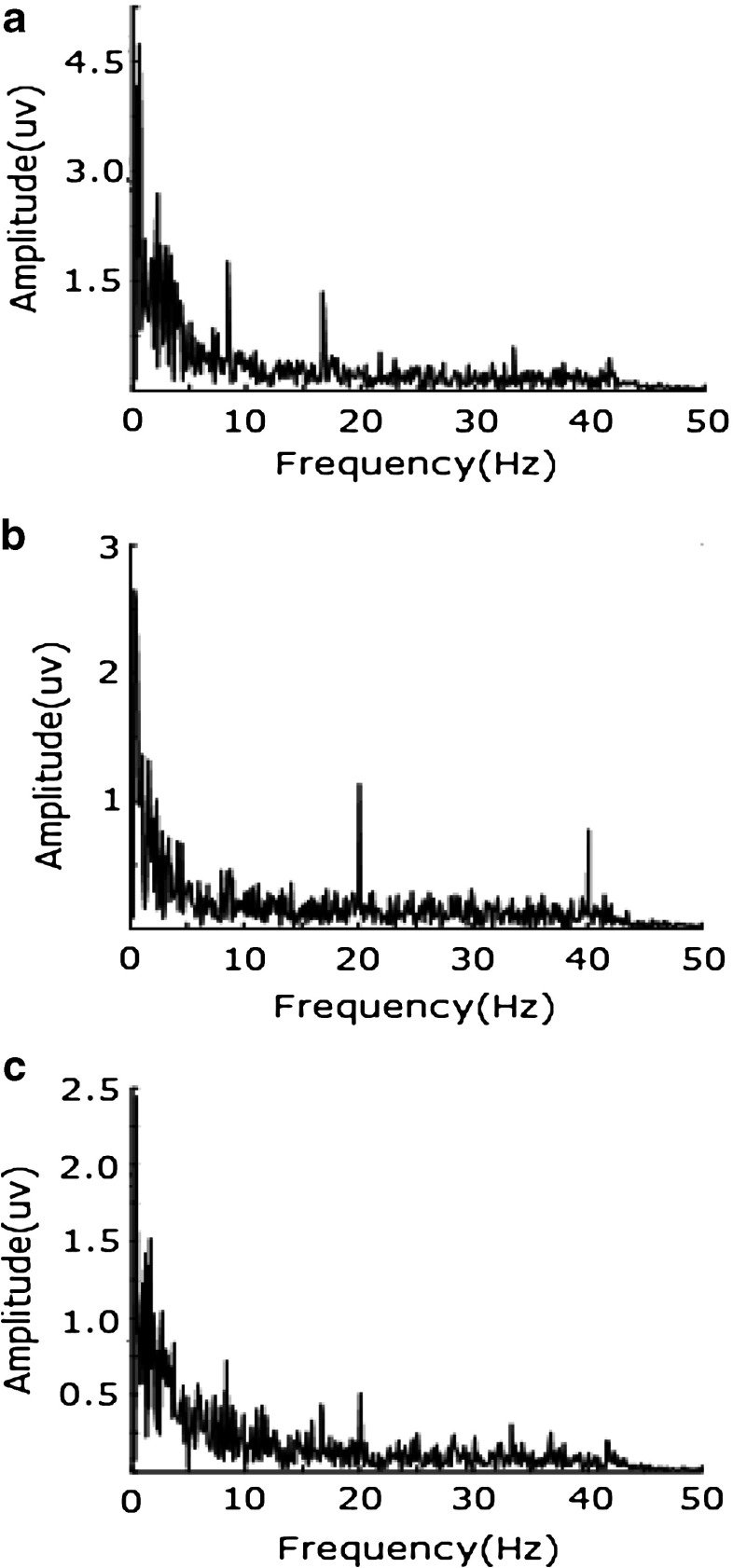
The spectrum of GZ’s SSVEP under different stimulus at electrode No. 60: **a** stimulated by 8.3-Hz flicker only; **b** stimulated by 20-Hz flicker only; **c** stimulated by 8.3-Hz and 20-Hz flickers simultaneously

In the first stage of a trial, there is no cognitive task, so the SSVEP in this period can be used to compare with that evoked separately. With most electrodes, a power of 8.3 Hz SSVEP is bigger than that of 20 Hz SSVEP either in the preliminary experiment or in the formal experiment, while the SSVEP power evoked in the formal experiment is smaller than that evoked in the preliminary experiment. Comparing the average SSVEP power of 8.3 Hz across all electrodes with that of 20 Hz, the ANOVA result across all subjects is (F(1, 18) = 4.49, *p* < 0.01) in the preliminary experiment and (F(1, 18) = 3.87, *p* = 0.01) in the formal experiment, which means that the power of 8.3 Hz is significantly bigger than that of 20 Hz, under situations of separate and simultaneous stimulation. The average SSVEP power of 8.3 Hz across all electrodes in the preliminary experiment is significantly bigger than that in the formal experiment (F(1, 18) = 9.25, *p* < 0.01), as is the power of 20-Hz SSVEP (F(1, 18) = 7.13, *p* < 0.01). As an example, Fig. 3 shows the results of subject GZ at electrode 60.

Although the power difference between different frequency SSVEPs is significant, the power distribution is similar and the power of 8.3-Hz and 20-Hz SSVEP are both concentrated at the occipital area. When stimulated separately, the average correlation coefficient across all subjects between 8.3-Hz and 20-Hz SSVEP power distribution is 0.89, and the ANOVA result across all subjects for the power distribution indicator between 8.3 and 20 Hz is (F(1, 18) = 0.17, *p* = 0.82), when stimulated simultaneously they are 0.93 and (F(1, 18) = 0.33, *p* = 0.47), respectively. This means that the distribution of different SSVEP neural networks is very similar.

The average correlation coefficient across all subjects of 8.3-Hz SSVEP power distribution between the separately and simultaneously stimulated is 0.95, the ANOVA result across all subjects for the power distribution indicator is (F(1, 18) = 0.24, *p* = 0.78). This means that the power distribution of 8.3-Hz SSVEP under the two situations is very similar, and the 8.3-Hz SSVEP neural network has not been affected by the 20-Hz SSVEP neural network.

The average correlation coefficient across all subjects of 20-Hz SSVEP power distribution between the separately and simultaneously stimulated is 0.96. The ANOVA result across all subjects for the power distribution indicator is (F(1, 18) = 0.21, *p* = 0.82). This means the power distribution of 20-Hz SSVEP under the two situations are also very similar and the 20-Hz SSVEP neural network has not been affected by the 8.3-Hz SSVEP neural network.

### The 8.3-Hz SSVEP power under a situation with cognitive tasks

**Fig. 4:**
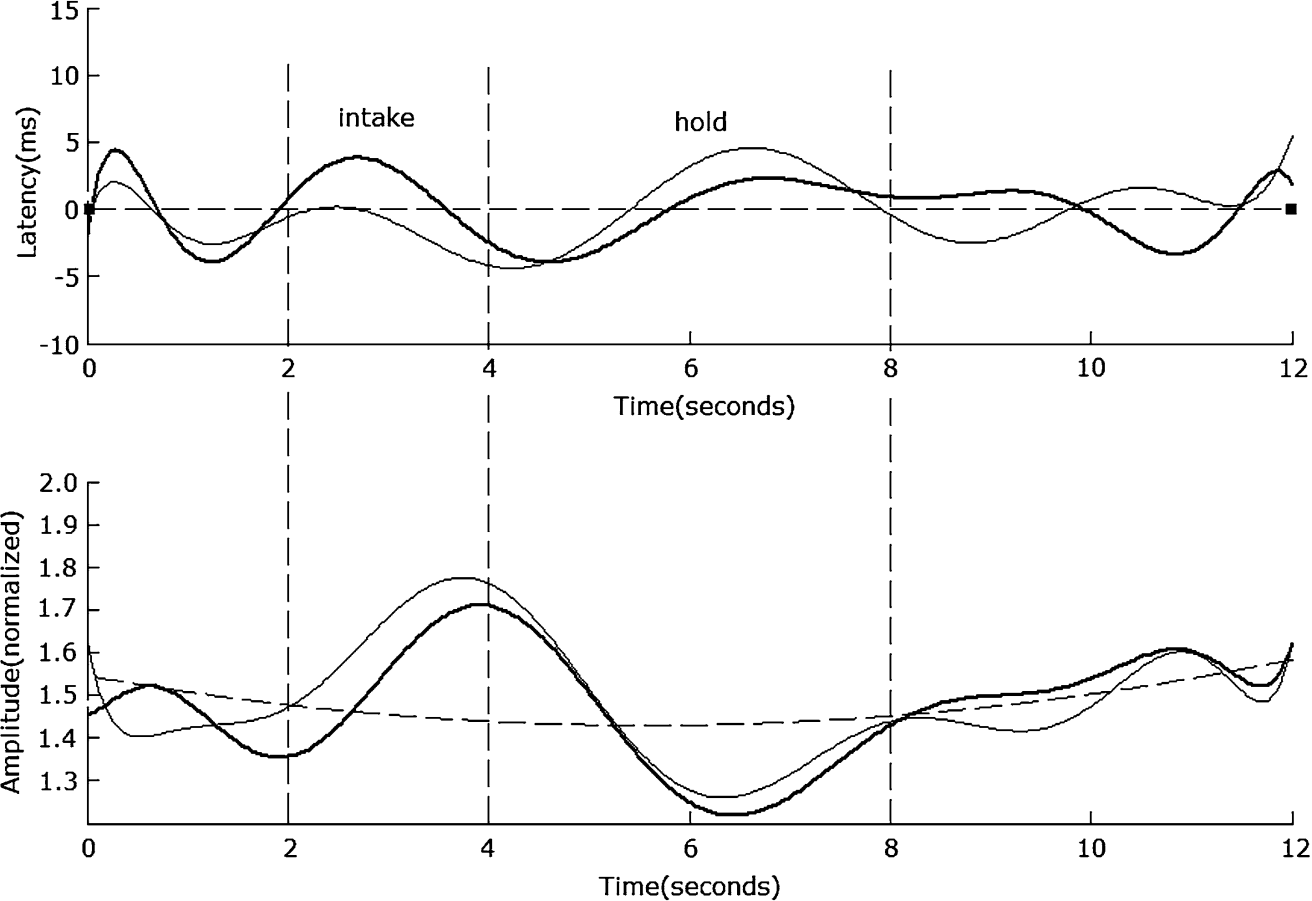
The 8.3-Hz SSVEP amplitude and latency at electrode No. 60. The upper traces illustrate the SSVEP latency difference between the C task and the LD task (*thin line*), the HD task (*bold line*), and the C task (*dotted line*), respectively. The lower traces illustrate the changes in the normalized SSVEP amplitudes, with the *thin line* for LD, the *bold line* for HD, and the *dotted line* for C

In the ‘prepare’ and ‘recover’ stage, the average normalized SSVEP amplitude across all electrodes and all subjects is very small, i.e., 0.63 for LD and 0.67 for HD. When comparing the average normalized SSVEP amplitude during these periods under LD task with that under HD task, the ANOVA result across all subjects is (F(1, 18) = 0.46, *p* = 0.53), which suggests that the intensity of brain activity during these periods is similar. With the ‘intake’ and ‘hold’ stages, the average normalized SSVEP amplitude across all electrodes and subjects is very clear, i.e., 1.45 for LD and 1.56 for HD. When comparing the average normalized SSVEP amplitude during these periods under LD task with that under HD task, the ANOVA result across all subjects is (F(1, 18) = 7.37, *p* = 0.04), which suggests that the intensity of brain activity during these periods under HD task is stronger than that under LD task. The average correlation coefficient across all electrodes and all subjects between HD and LD modulated SSVEP amplitude waveforms is 0.88. As an example, Fig. 4 shows the waveform of 8.3 Hz modulated SSVEP at No. 60 electrode in the formal experiment.

### The 20-Hz SSVEP power under a situation with cognitive tasks

**Fig. 5:**
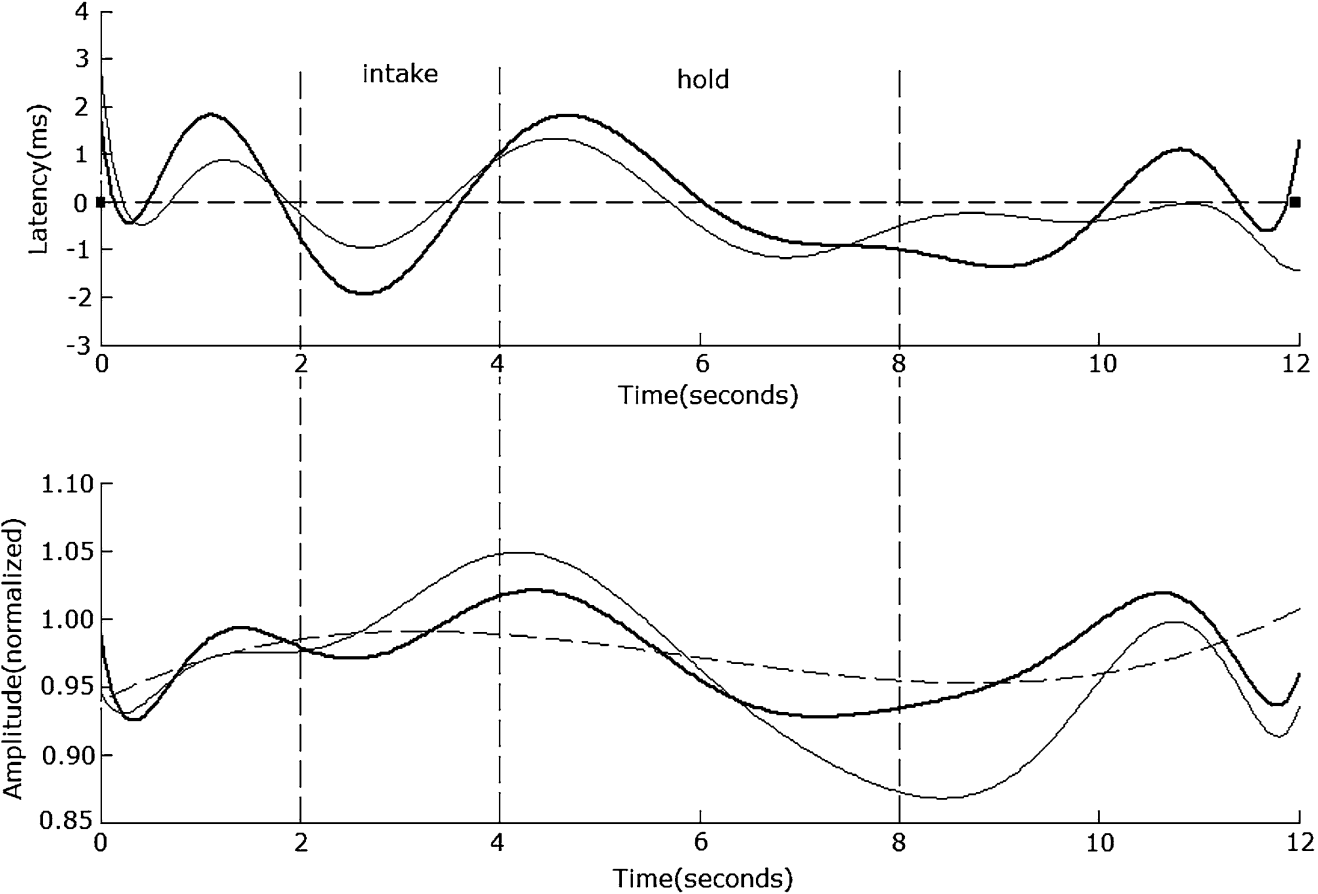
The 20-Hz SSVEP amplitude and latency at electrode No. 60. The upper traces illustrate the SSVEP latency difference between the C task and the LD task (*thin line*), the HD task (*bold line*), and the C task (*dotted line*). The lower traces illustrate the changes in the normalized SSVEP amplitudes, with the *thin line* for LD, the *bold line* for HD, and the *dotted line* for C

The 20-Hz SSVEP property in different stages is very similar to that of 8.3 Hz. In the ‘prepare’ and ‘recover’ stages, the average normalized amplitude across all electrodes and all subjects is 0.51 for LD and 0.55 for HD. When comparing the average normalized SSVEP amplitude during these periods under LD task with that under HD task, the ANOVA result across all subjects is (F(1, 18) = 0.39, *p* = 0.71), which suggests that the intensity of brain activity during these periods is similar to each other. In the ‘intake’ and ‘hold’ stages, the average normalized amplitude across all electrodes and all subjects is 1.15 for LD and 1.28 for HD. When comparing the average normalized SSVEP amplitude during these periods under LD task with that under HD task, the ANOVA result across all subjects is (F(1, 18) = 5.42, *p* = 0.038), which suggests that the intensity of brain activity during these periods under HD task is stronger than that under LD task. The average correlation coefficient across all electrodes and all subjects between HD and LD modulated SSVEP amplitude waveforms is 0.82. As an example, Fig. 5 shows the 20-Hz modulated SSVEP waveform at electrode No. 60 in the formal experiment. The trend in each stage is very similar to that of 8.3 Hz shown in Fig. 4.

### The normalized SSVEP distribution under a situation with cognitive tasks

**Fig. 6:**
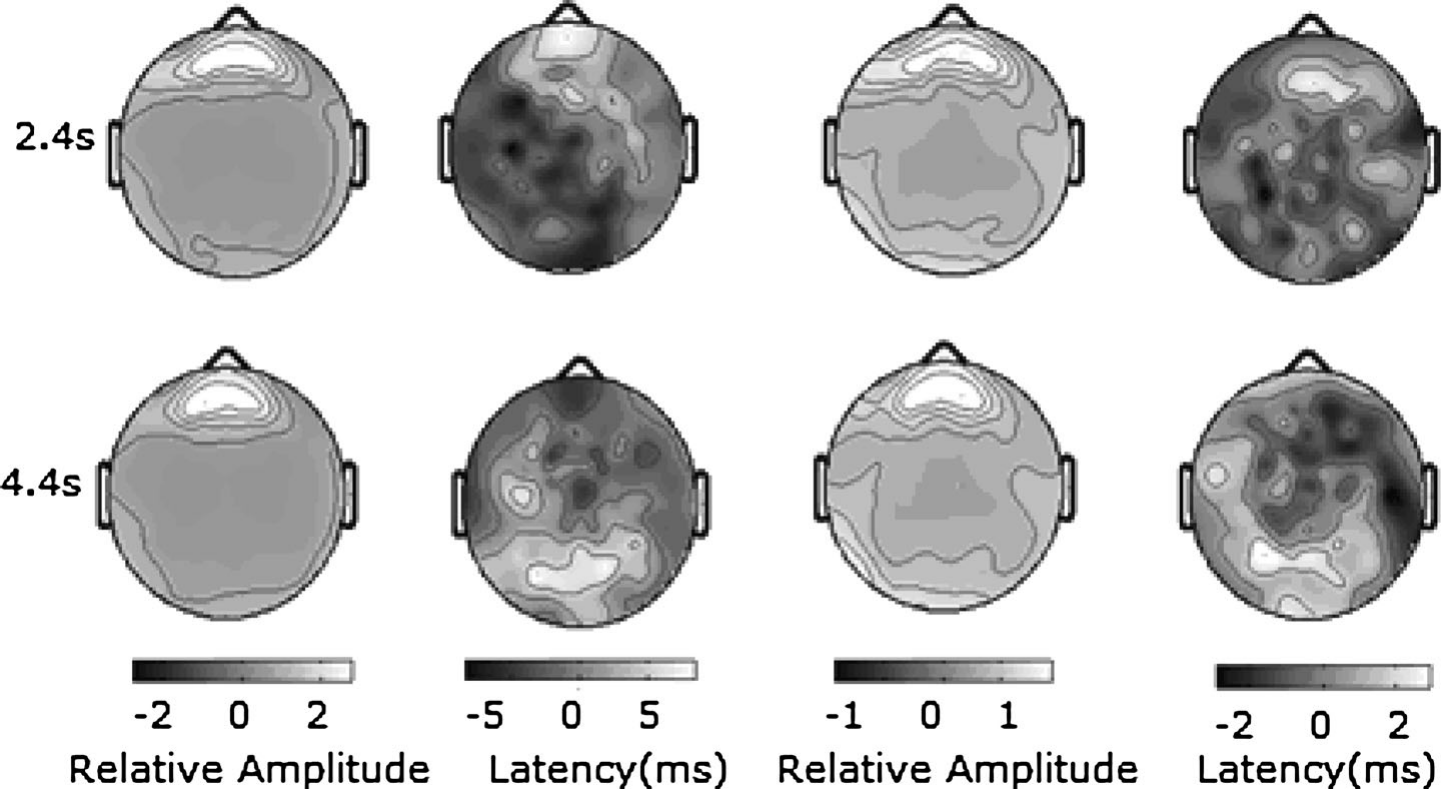
Topographic maps of different-frequency SSVEP at different stages when the subjects perform the HD task. The two *left columns* are that of 8.3-Hz SSVEP (the left one is for normalized amplitude and the right one is for latency); the *top* is obtained during the ‘intake’ period (2.4 s after the beginning of a trial) and the *bottom* is obtained during the ‘hold’ period (4.4 s after the beginning of a trial). The two right columns are for the 20-Hz SSVEP

The distributions of 8.3-Hz and 20-Hz normalized SSVEP in the formal experiment are similar. This can be seen from the brain topographic map qualitatively, while the correlation coefficient between 8.3 and 20 Hz normalized SSVEP distribution exhibits this similarity quantitatively. At the beginning of the ‘intake’ stage, for example, 2.4 s after the beginning of a trial, the average correlation coefficient between 8.3- and 20-Hz normalized SSVEP distribution across all subjects is 0.85 for HD and 0.87 for LD. The ANOVA result across all subjects for the power distribution indicator between 8.3- and 20-Hz normalized SSVEP is (F(1, 18) = 0.34, *p* = 0.25) for HD and (F(1, 18) = 0.28, *p* = 0.31) for LD. This means that the power modulation on different SSVEP neural networks by a cognitive task is similar. At the beginning of the ‘hold’ stage, for example, 4.4 s after the beginning of a trial, the average correlation coefficient between 8.3 and 20 Hz normalized SSVEP distribution is 0.82 for HD and 0.78 for LD. The ANOVA result across all subjects for the power distribution indicator between 8.3- and 20-Hz normalized SSVEP is (F(1, 18) = 0.15, *p* = 0.52) for HD and (1, 18) = 0.34, *p* = 0.25) for HD and ((1, 18) = 0.28, *p* = 0.31) for LD. This suggests that the power modulation on different SSVEP neural networks by the cognitive task is similar during this stage. The averaged correlation coefficient between the modulated SSVEP waves of 8.3 and 20 Hz across all electrodes and all subjects is 0.87 for HD and 0.89 for LD in this work, while it was 0.72 for HD and 0.7 for LD in our previous work. The ANOVA result across all subjects for the average correlation coefficient between the modulated SSVEP waves of 8.3 and 20 Hz for all electrodes is (*F*(1, 18)= 6.23, p < 0.01) for HD and (*F*(1, 18) = 4.58, p < 0.01) for LD. This means that the similarity between modulated SSVEP waves improves significantly when SSVEP neural networks are activated simultaneously. However, the latency distributions are very distinct even in a short period, for which we have not conducted a detailed investigation. Figure 6 shows the SSVEP amplitude and latency distribution under the HD task.

## Discussion

The aim of this work was to study the similarity between modulated SSVEPs when SSVEP neural networks are activated simultaneously and following this to understand the possibility of applying SSVEP in more wide frequency bands in order to study the process of a cognitive task. The detailed process of the working memory task modulation on SSVEP neural networks has been discussed in similar experiments [10,26], so the discussion related to the SSVEP process itself will not be repeated here.

In both the preliminary experiment and the formal experiment, the power of 20-Hz SSVEP is smaller than that of 8.3-Hz SSVEP and this is consistent with the results in other works [2,9]. 8.3-Hz and 20-Hz SSVEP are generated by different neural networks and a resonance peak of 8.3-Hz network is higher than that of a 20-Hz network. When the different SSVEP neural networks are activated simultaneously, the modulation depth of each flicker decreases compared with that when the networks are activated separately. Because the SSVEP power is positively correlative to the modulation depth of the flicker, SSVEP power decreases too. The power distribution for different SSVEP neural networks is similar to each other when the networks are activated separately and this can be seen from the correlation coefficient and the ANOVA result. When the SSVEP neural networks are activated simultaneously, they hold the same trend and the power distribution is similar to that when activated separately. This means that SSVEP neural network distribution is similar and independent to a large extent. Even when activated simultaneously, there is no interaction between them or the interaction can be neglected. This can promise modulation on certain SSVEP neural networks coming from the cognitive task, but not from other SSVEP neural networks. Furthermore, because the modulation curve shows the relative amplitude between HD and C tasks or between LD and C tasks, SSVEP power decreases when the SSVEP neural networks are activated simultaneously having no effect on the modulation curve.

Based on EEG or MRI, many studies have demonstrated that different brain areas may be activated when the subject executes different cognitive tasks [3]. In other words, there surely exist different cognitive neural networks. It is very difficult to confirm whether the cognitive neural networks and the SSVEP neural networks have some physical overlap. However, it is demonstrated that there is some interaction between these two types of neural network. In one of our studies, we found that the cognitive task can modulate the amplitude of SSVEP by its ERP [27], and the SSVEP can be seen as a carrier, while the ERP can be seen as a modulation signal. Many works have employed the method of studying the process of a cognitive task based on the SSVEP of a fixed frequency. The premise to popularize this method to a more wide frequency band SSVEP rests on the interaction between the cognitive neural network and the SSVEP neural network being stable, requiring modulation on different frequency SSVEP by the same ERP with consistency to a large extent. If not the case, it is unreasonable to combine SSVEP variance with the process of a cognitive task. In order to check this consistency, in 2007 we used two flickers with different frequencies to activate the different SSVEP neural networks separately and asked the subjects to accomplish a working memory task at the same time [26]. The results showed that the modulation on different SSVEP neural networks possess some consistencies, but sometimes there is a big difference. This discrepancy may be because of the different background noise when the SSVEP neural works are activated separately, so we redesigned the experiment in this work. The results show that modulated SSVEP waves are more similar to each other than previously indicated and that modulated SSVEP power distributions are more alike in the key time periods.

The improved similarity of the modulated SSVEP waves and power distribution can be explained via the following reasons. The first reason is the variance of SSVEP itself. As we know, the components in EEG distribute in a wide frequency band. When stimulated by a repetitive flicker, components the same as the stimulus frequency can be strengthened, so a clear peak can be observed in the EEG spectrum. The SSVEP can be seen as constructed from two parts: one from the potential brain activity, and the other from the repetitive stimulus [1,28]. The first part usually changes because potential brain activities vary in different periods. Based on the theory of limit cycle oscillations, the second part can be taken as stable. When studying modulation on different SSVEP neural networks with the same cognitive task, if the SSVEP neural networks are activated separately, the influence on carriers by the background noise is quite different from the situation in which two SSVEP neural networks are activated simultaneously and this results in more error in terms of the modulated SSVEP waveforms.

The second reason is the variance of ERP itself. The most popular viewpoint is that the ERP of a cognitive task in a short time period is the same, so we can use the superposition method to cancel the background noise and get the ERP. However, for a cognitive task of long duration, the ERP itself may have some variance for some other irrelevant psychological activity. For the cognitive task in this work, the ‘hold’ stage is 4 s and the subjects can distract their attention during this period. For example, they can imagine other things if they think they have memorized the features. In this situation, even the carrier holds unvaried, the modulation signal (ERP) has some minor differences and this produces some errors in the modulated SSVEP waveforms. When the SSVEP neural networks are activated simultaneously, the error of the modulation signal decreases, and this makes the error of the modulated SSVEP waves smaller than those activated separately.

In this experiment, in the ‘intake’ stage and the early period of the ‘hold’ stage, the modulation on different SSVEP neural networks by a cognitive task is more consistent. In the 2-s ‘intake’ stage, the subjects try to understand the features of the stimulus; normally they would not distract their attention, so the ERP in this period is stable. At the beginning of the ‘hold’ stage, the subjects try to remember the features, so they must not distract their attention, and the ERP in this period is also stable. This makes the modulated SSVEP waves in these periods have a high similarity between different tasks or between different SSVEP neural networks. This is also the case for the modulated SSVEP power distribution. In other periods, the subjects may distract their attention or imagine other things and this makes the ERP vary, thus the similarity between modulated SSVEPs decreases. Therefore, when using SSVEP to study a cognitive task of long duration, great care is needed to explain the variance of SSVEP and more emphasis needs to be placed on key time periods, but not all time periods.

Since the SSPT method was introduced to study cognitive tasks of a long duration, many works based on the SSPT have been done. In most of these studies, the authors used a 13-Hz flicker for evoking SSVEP [4,5,10,17,19,20]. Few people have discussed the validity of SSPT on other frequency bands, and this limits the use of the SSPT method. From this work, it shows that a cognitive task can modulate the SSVEP in different frequency bands with a similar style. Therefore, when using different frequency SSVEP as a carrier to study the process of a cognitive task of long duration, the SSVEP amplitude variance can be explained in the same way, and this can expand the application of the SSPT method. On the other hand, although the modulation wave for different frequency SSVEP by the same cognitive task is similar, modulation on the low-frequency SSVEP is deeper than that on the high-frequency SSVEP, and this can be seen from the normalized SSVEP amplitude. Therefore, in order to understand the details of the process more clearly, it is reasonable to select a low-frequency SSVEP as the carrier when studying the processes of a recognitive task.

## Conclusions

Different frequency SSVEP neural networks can be activated simultaneously and modulated by a cognitive task with a similar style, while the interaction between SSVEP neural networks can be neglected. The similarity of the modulated SSVEPs under this situation is higher than when evoked separately. The results of this work suggest that SSVEP in different neural networks can be legitimately used to study cognitive processes, i.e., the SSPT method can be popularized to a more wide frequency band and some time periods are key to explaining the process of a cognitive task.
